# *ExRec*: a python pipeline for generating recombination-filtered multi-locus datasets

**DOI:** 10.1093/bioadv/vbad174

**Published:** 2023-11-29

**Authors:** Sam McCarthy Potter, W Bryan Jennings

**Affiliations:** Department of Biology, Carleton College, Northfield, MN 55057, United States; Department of Evolution, Ecology & Organismal Biology, University of California, Riverside, CA 92521, United States

## Abstract

**Summary:**

*ExRec* (Exclusion of Recombined DNA) is a dependency-free Python pipeline that implements the four-gamete test to automatically filter out recombined DNA blocks from thousands of DNA sequence loci. This procedure helps all loci better meet the “no intralocus recombination” assumption common to many coalescent-based analyses in population genomic, phylogeographic, and shallow-scale phylogenomic studies. The user-friendly pipeline contains five standalone applications—four file conversion scripts and one main script that performs the recombination filtering procedures. The pipeline outputs recombination-filtered data in a variety of common formats and a tab-delimited table that displays descriptive statistics for all loci and the analysis results. A novel feature of this software is that the user can select whether to output the longest nonrecombined sequence blocks from recombined loci (current best practice) or randomly select nonrecombined blocks from loci (a newer approach). We tested *ExRec* with six published phylogenomic datasets that ranged in size from 27 to 2237 loci and came in a variety of input file formats. In all trials the data could be easily analyzed in only seconds for the smaller datasets and <30 min for the largest using a simple laptop computer.

**Availability and implementation:**

*ExRec* was written in Python 3 under the MIT license. The program applications, user manual (including step-by-step tutorials), and sample data are freely available at https://github.com/Sammccarthypotter/ExRec.

## 1 Introduction

The most widely used multispecies coalescent (MSC) model in phylogenomic studies assumes that there has been no recombination within each DNA sequence locus since the most-recent common ancestor to the sampled haplotype sequences (Felsenstein 2004, Degnan and Rosenberg 2009, Edwards 2009, Jennings 2016, Bravo *et al.* 2019, Rannala *et al.* 2020). But how much of a concern is the no intralocus recombination assumption in practice? Zhu and Yang (2021) recently voiced that this assumption “is of particular concern,” however it is not yet clear if violations of this assumption greatly impact MSC parameter estimates or not. Indeed, some simulation-based studies found that violating this assumption did not adversely affect species tree and/or historical demographic parameter inferences (e.g. Lanier and Knowles 2012, Zhu *et al.* 2022, Yan *et al.* 2023), while other studies obtained contradictory evidence (e.g. Strasburg and Rieseberg 2008, 2010, Hey and Wang 2019, Hill and Roch 2022). Thus, until this debate is resolved, researchers should have access to bioinformatics tools that can easily “filter out” recombined DNA sequences from multi-locus data, keeping with current best practice.

Hey and Nielsen (2004) suggested a procedure to filter out recombined blocks of DNA sequences from multi-locus datasets. Their method uses the four-gamete test (Hudson and Kaplan 1985) to identify presumably recombined sequence blocks, which can then be excised to leave the longest nonrecombined blocks for use in MSC-based analyses. Hey and Wang (2019) labeled this method “four-gamete filtering.” A minimum of four phased haplotypes are required to conduct this test. An important assumption of the four-gamete test is that each nucleotide site can undergo a maximum of one base substitution (i.e. infinite sites model).

In a simulation study, Hey and Wang (2019) concluded that four gamete filtering can improve estimates of some MSC parameters (compared to using nonfiltered data), but they also discovered that the “longest block” approach can yield biased estimates for some parameters. An alternative approach suggested by these workers is to randomly select nonrecombined blocks instead of the longest blocks. When they applied the “random blocks” approach to simulated data they found that some parameter estimates were more accurate than estimates obtained using the longest blocks method—but the former approach was less powerful than the latter. Until this problem is studied more thoroughly, researchers should conduct both types of analyses to see how their results vary. Here, we present *ExRec* (Exclusion of Recombined DNA), a user-friendly Python package that uses both longest and random blocks approaches to automatically filter out recombined sequences from multi-locus data and outputs the filtered data in a variety of common formats.

## 2 *ExRec* pipeline

**Figure 1.**
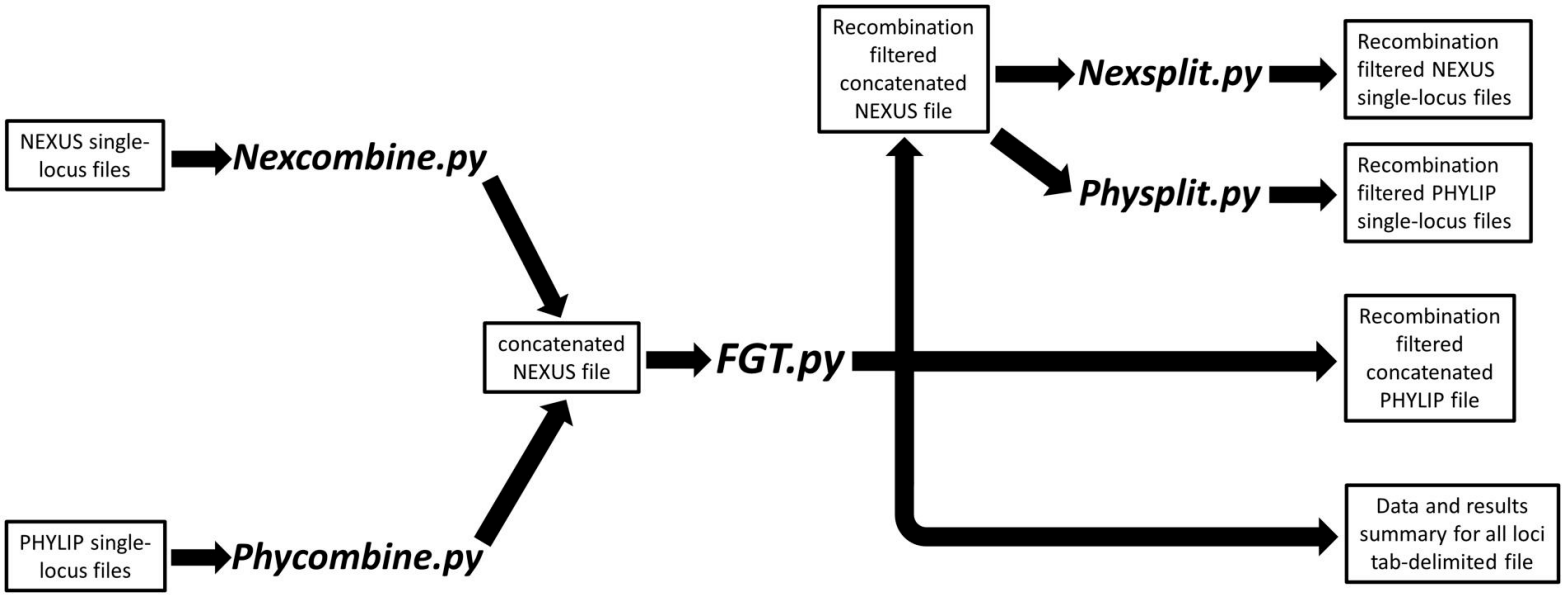
*ExRec* software pipeline. Text boxes show input and output files while the stand-alone Python 3 applications are in bold italic letters.

The *ExRec* package contains five stand-alone Python applications, which form a user-friendly pipeline to filter away recombined blocks of DNA sites from hundreds to thousands of DNA sequence loci (Fig. 1). In addition to a user manual that includes step-by-step tutorials, the package also includes two example datasets. The user executes each application from the command line and a help command is available in each application that shows step-by-step instructions in concise form.

The main application, *FGT.py*, implements the four-gamete test to filter away presumably recombined blocks of sites from each locus, and then outputs the filtered data in multi-locus NEXUS and PHYLIP file formats as well as a tabular summary of the data and results that can be copy and pasted into a spreadsheet for meta-analyses (Fig. 1). The user can have *FGT.py* output the longest or randomly selected nonrecombined blocks for each locus that contains evidence of historical recombination. The summary table contains descriptive statistics about each locus and results of the four-gamete filtering analyses including: locus name, locus starting length (base pairs [bp]), length (bp) excluding gaps/missing data at sites, S (number of segregating sites), list of sites that violate the infinite sites model, *R_M_* (minimum number of recombination events; Hudson and Kaplan 1985), pairs of sites that had recombination event(s) within them, sites that define the longest nonrecombined block, and the length of the retained longest (or randomly selected) nonrecombined block. We designed *FGT.py* to output identical descriptive statistics for each locus and results of four-gamete tests that are produced by the program *DNAsp* version 6 (Rozas *et al.* 2017)—a longtime standard DNA analysis tool in the community. The required input file for *FGT.py* is a concatenated partitioned interleaved NEXUS file. Because of the somewhat “big data” complexity of this large NEXUS file, a file that can contains hundreds to thousands of DNA sequence loci, we provide the user with two file conversion applications in this package, *Nexcombine.py* and *Phycombine.py*, which convert single-locus (sequential or interleaved) NEXUS or PHYLIP files, respectively, into a concatenated NEXUS file that is ready for input into *FGT.py* (Fig. 1).

After running *FGT.py*, the user can input the filtered multi-locus data files into species tree programs such as *NJst* (Liu and Yu 2011) or in species delimitation/historical demography programs like the *Bayesian Phylogenetics and Phylogeography* (*BPP*) software program (Yang 2015, Flouri *et al.* 2018). Alternatively, the user can use the applications *Nexsplit.py* or *Physplit.py* in the package to separate the recombination-filtered data into single-locus NEXUS or PHYLIP files, respectively (Fig. 1). The single-locus files can then be batch-input into phylogenetic programs prior to conducting summary methods species tree analyses such as can be implemented in the software *ASTRAL* (Mirarab *et al.* 2014, 2016).

It is important to emphasize that owing to the infinite sites model assumption for the four-gamete test, it is not appropriate to use *ExRec* to analyze datasets for monophyletic groups containing ancient divergences because the model would be badly violated, and thus cause *FGT.py* to improperly filter datasets. Similarly, datasets containing many questionable base calls should also not be analyzed with *ExRec* because base call errors and artifactual sequence gaps (false positive indels) will also lead to spurious results. Users should also ensure that their datasets contain phased sequences, which can be obtained from second generation (Illumina) DNA sequencing data via the use of bioinformatics tools such as *SECAPR* (Andermann *et al.* 2018) and *PHYLUCE* (Faircloth 2016) or directly from long-read sequencing platforms like PacBio and Oxford Nanopore.

To the best of our knowledge, there are only two published software packages that can conduct four-gamete filtering of multi-locus data. The first program, *Imgc* (Woerner *et al.* 2007), requires input sequence data to be aligned FASTA files, whereas a more recent program called *Pop-Gen Pipeline Platform* (*PPP*; Webb *et al.* 2021) requires input data to be in vcf file format. We chose the data input formats for *ExRec* to be NEXUS or PHYLIP because these two formats are among the most popular formats for single-locus multiple sequence alignments in phylogenomics. *Imgc* outputs filtered data in aligned FASTA and IM file formats, while *PPP* outputs filtered data in vcf, Ima3, Gphocs, fastsimcoal, dadi, PED, and treemix formats. In contrast, we designed *ExRec* to output filtered data in single and multi-locus NEXUS and PHYLIP formats because these formats are usually the required inputs for summary species tree analyses and some historical demography software packages such as *BPP* (Yang 2015). Despite the widespread use of NEXUS and PHYLIP files in phylogenomic studies, we acknowledge that one limitation of *ExRec* is that it does not output recombination-filtered data in other formats such as vcf and IM formats. However, file conversion programs that can convert NEXUS and PHYLIP files into these and other formats are available (e.g. *PGDSpider*, Lischer and Excoffier 2012).

## 3 Test datasets and benchmarking

To validate the *ExRec* pipeline and evaluate its versatility, we tested it with 6 different published phylogenomic datasets: 27 anonymous nuclear loci or “ALs” for Australian grass finches (Jennings and Edwards 2005), 292 ALs for hominoids (Costa *et al.* 2016), 70 ultraconserved elements or “UCEs” for plethodontid salamanders (Newman and Austin 2016), 47 UCEs for owls (Salter *et al.* 2020), 2237 UCEs for manakin birds (Leite *et al.* 2021), and 465 UCEs for Middle American cichlid fishes (Alda *et al.* 2021). The data from these studies came in various formats including sequential and interleaved NEXUS and PHYLIP formats. This is important because many different variants of NEXUS and PHYLIP files are used in phylogenomics studies. It is therefore essential for DNA sequence analysis programs to show little or no specificity for different input file variants (i.e. accepting some while rejecting others). We designed the *Nexcombine.py* and *Phycombine.py* applications to accept NEXUS and PHYLIP files, respectively, in either sequential or interleaved formats. Moreover, *Phycombine.py* can process a range of PHYLIP file variants, which includes (at least) four different strict sequential formats, two strict interleaved formats, two relaxed-name sequential formats, and two relaxed-name interleaved formats (see user manual for more details). We therefore wanted to determine if the *ExRec* pipeline could handle diverse input file types from published studies. Next, we compared the descriptive statistics for each locus, *R_M_* estimates, and inferred locations of recombination events generated by *FGT.py* to comparable values produced using *DNAsp* version 6 (Rozas *et al.* 2017). Lastly, we tested the random block option in *FGT.py* by running the application twenty independent times on the 27-locus finch dataset to determine if loci containing more than one nonrecombined sequence block were randomly chosen. All analyses were performed on a Samsung Book laptop with an Intel Core i5-1135G7 processor @2.40 GHz, and Windows 10. Runtimes were recorded for each *ExRe*c application trial.

## 4 Results

### 4.1 Initial data input step

The six datasets we analyzed with the *ExRec* pipeline were in two different NEXUS and two different PHYLIP formats (Table 1). We did not encounter any problems while executing the *Nexcombine.py* and *Phycombine.py* applications to convert the NEXUS and PHYLIP files, respectively, into the required concatenated partitioned interleaved NEXUS file for the main program, *FGT.py* (Fig. 1). These file conversion applications processed the smallest and largest datasets in 1 s and 1 min, respectively (Table 1).

**Table 1.** Example computer runtimes for the five *ExRec* application scripts using six published phylogenomic datasets.^a^

Study	Loci	Number of Loci (Sequences/locus)	File format	Running time (minutes:seconds)				
				Nexcombine	Phycombine	FGT	Nexsplit	Physplit
Jennings and Edwards (2005)	AL	27 (4)	Nexus (seq)	00:01	na	00:02	00:01	00:01
Costa *et al.* (2016)	AL	292 (4)	Nexus (inter)	00:05	na	00:33	00:02	00:02
Newman and Austin (2016)	UCE	70 (40*)	Nexus (seq)	00:03	na	03:08	00:02	00:02
Leite *et al.* (2021)	UCE	2237 (54*)	Nexus (seq)	01:00	na	22:20	00:42	00:30
Salter *et al.* (2020)	UCE	47 (43*)	Phylip (inter)	na	00:02	00:17	00:02	00:02
Alda *et al.* (2021)	UCE	465 (93*)	Phylip (seq)	na	00:23	08:32	00:10	00:07

a AL, anonymous nuclear loci; UCE, ultraconserved elements loci; seq, sequential format; inter, interleaved format; na, not applicable; *, approximate number of sequences per locus owing to unequal sample sizes.

### 4.2 Four-gamete filtering of each dataset

Using the output file (i.e. concatenated partitioned interleaved NEXUS file) obtained from each of the *Nexcombine.py* and *Phycombine.py* file conversion runs we then input these files into the main application, *FGT.py* (Fig. 1). *FGT.py* required two seconds to conduct the recombination filtering procedures on the smallest dataset, whereas it needed 22 min to finish the largest one (Table 1). In all analyses, the descriptive statistics for each locus, *R_M_* estimates, inferred locations of recombination events generated by *FGT.py* were identical to values output by *DNAsp* version 6 (Rozas *et al.* 2017). These results show that *FGT.py* can automatically conduct recombination-filtering procedures on at least thousands of DNA sequence loci in less than a half hour on a simple laptop computer.

When we evaluated the ability of *FGT.py* to randomly choose nonrecombined sequence blocks, we found, as expected, that the application selected each block in ∼50% of the analysis runs from the six loci having two nonrecombined blocks (i.e. loci Pa_11, Pa_13, Pa_20, Pa_24, Pa_26, and Pa_29). These results confirm that *FGT.py*, when in random-block mode, randomly selects nonrecombined blocks.

### 4.3 Creating recombination-filtered single-locus files

We retrieved the recombination-filtered multi-locus NEXUS files output from the *FGT.py* runs with the six test datasets and easily converted them into single-locus NEXUS and PHYLIP files using the applications *Nexsplit.py* and *Physplit.py*. Runtimes for *Nexsplit.py* ranged from 1–42 s for the smallest and largest datasets, respectively, whereas runtimes for *Physplit.py* ranged from 1–30 s for the two datasets, respectively (Table 1).

## 5 Conclusion

In summary, *ExRec* is a versatile user-friendly software pipeline that can automatically generate recombination-filtered data in a variety of file formats in seconds to minutes depending on the size of the input dataset. The critical first step of the pipeline is robust to different variants of NEXUS and PHYLIP files making it trouble-free to operate. We believe that the *ExRe*c pipeline can play an important role in helping the phylogenomics community resolve the ongoing debate about the importance of the no intralocus recombination assumption in MSC-based population genomic, phylogeographic, and shallow-scale phylogenomic studies.

## Data Availability

The data used in the paper are publicly available and the sources have been cited in the text, except for the original unfiltered Australian grass finch data, which can be accessed at https://github.com/Sammccarthypotter/ExRec.
